# Time to reconcile research findings and clinical practice on upper limb neurorehabilitation

**DOI:** 10.3389/fneur.2022.939748

**Published:** 2022-07-19

**Authors:** Leonardo Boccuni, Lucio Marinelli, Carlo Trompetto, Alvaro Pascual-Leone, José María Tormos Muñoz

**Affiliations:** ^1^Institut Guttmann, Institut Universitari de Neurorehabilitació adscrit a la UAB, Badalona, Spain; ^2^Universitat Autònoma de Barcelona, Bellaterra, Spain; ^3^Fundació Institut d'Investigació en Ciències de la Salut Germans Trias i Pujol, Badalona, Spain; ^4^Department of Neuroscience, Rehabilitation, Ophthalmology, Genetics, Maternal and Child Health, University of Genova, Genova, Italy; ^5^IRCCS Ospedale Policlinico San Martino, Department of Neuroscience, Division of Clinical Neurophysiology, Genova, Italy; ^6^IRCCS Ospedale Policlinico San Martino, Department of Neuroscience, Division of Neurorehabilitation, Genova, Italy; ^7^Hinda and Arthur Marcus Institute for Aging Research and Deanna and Sidney Wolk Center for Memory Health, Hebrew SeniorLife, Boston, MA, United States; ^8^Department of Neurology and Harvard Medical School, Boston, MA, United States

**Keywords:** stroke, neurorehabilitation, upper limb, motor learning, personalized medicine

## Abstract

**The problem:**

In the field of upper limb neurorehabilitation, the translation from research findings to clinical practice remains troublesome. Patients are not receiving treatments based on the best available evidence. There are certainly multiple reasons to account for this issue, including the power of habit over innovation, subjective beliefs over objective results. We need to take a step forward, by looking at most important results from randomized controlled trials, and then identify key active ingredients that determined the success of interventions. On the other hand, we need to recognize those specific categories of patients having the greatest benefit from each intervention, and why. The aim is to reach the ability to design a neurorehabilitation program based on motor learning principles with established clinical efficacy and tailored for specific patient's needs.

**Proposed solutions:**

The objective of the present manuscript is to facilitate the translation of research findings to clinical practice. Starting from a literature review of selected neurorehabilitation approaches, for each intervention the following elements were highlighted: definition of active ingredients; identification of underlying motor learning principles and neural mechanisms of recovery; inferences from research findings; and recommendations for clinical practice. Furthermore, we included a dedicated chapter on the importance of a comprehensive assessment (objective impairments and patient's perspective) to design personalized and effective neurorehabilitation interventions.

**Conclusions:**

It's time to reconcile research findings with clinical practice. Evidence from literature is consistently showing that neurological patients improve upper limb function, when core strategies based on motor learning principles are applied. To this end, practical take-home messages in the concluding section are provided, focusing on the importance of graded task practice, high number of repetitions, interventions tailored to patient's goals and expectations, solutions to increase and distribute therapy beyond the formal patient-therapist session, and how to integrate different interventions to maximize upper limb motor outcomes. We hope that this manuscript will serve as starting point to fill the gap between theory and practice in upper limb neurorehabilitation, and as a practical tool to leverage the positive impact of clinicians on patients' recovery.

## Introduction

There is a translational gap between research findings and clinical practice in the field of upper limb neurorehabilitation. The latest decades have seen an exponential growth of evidence, that has not been followed by an adequate evolution of treatments offered to patients. One example is Constraint-Induced Movement Therapy (CIMT), an intensive, motor-learning based intervention that has demonstrated the highest evidence level for the improvement of upper limb motor function (1), with results successfully replicated in several randomized controlled trials (2). Despite two decades of consistent findings, CIMT is rarely applied in most clinical centers, whereas other interventions like Neurodevelopmental Treatment (NDT, also known as Bobath concept) are popular among therapists, irrespective that research evidence has shown equal or even inferior effectiveness for NDT, in comparison with other approaches (1, 3, 4). There are likely multiple reasons to account for this. Sometimes adoption of a certain intervention in the clinical practice flow is challenging because of time, resources, training, and other requirements. Other times, the scientific evidence is difficult to apply to the individual patient since studies often focus on group average effects effectively disregarding – rather than leveraging – individual variability. The kind of personalized, optimal effective treatment we all desire and that our patients deserve requires overcoming these challenges.

A first step toward evidence-based practice is the identification, within each intervention, of those core elements (active ingredients) that are essential for treatment effectiveness (5). For motor training approaches, active ingredients could be for instance relative to the organization of task practice (number of repetitions, variability, global vs. selective movement practice), the provision of a certain type of feedback (implicit vs. explicit feedback), and so on. The following step is the identification of motor learning principles and neuroplastic mechanisms underlying recovery, to interpret the theoretical basis and the concrete results of any neurorehabilitation approach. We could start by considering four general motor learning principles: use-dependent learning (6–8), reinforcement learning (9, 10), explicit strategies (11, 12), and implicit recalibration (13, 14). To make an example with a reaching task, performance will show higher improvements for frequently reached targets with stable conditions, in comparison to rarely reached target (use-dependent learning), or when there is excessive variability of conditions (reinforcement learning). The person being trained is aware of the goals of the training based on instructions received (explicit strategies), but unaware of the kinematics and automatic adjustments underlying movement performance (implicit recalibration). A broader classification of motor learning principles related to neurorehabilitation have been proposed by Maier et al. in 2019, specifically by pointing out the following 15 principles: Repetitive practice, spaced practice, dosage, task-specific practice, goal-oriented practice, variable practice, increasing difficulty, multisensory stimulation, rhythmic cueing, explicit feedback/knowledge of results, implicit feedback/knowledge of performance, modulate effector selection, action observation/embodied practice, motor imagery, and social interaction (15). Given their practical utility to describe neurorehabilitation interventions, we will refer to these principles throughout the manuscript. Regarding neuroplastic mechanisms, we should at least distinguish two models of brain reorganization after stroke, the *interhemispheric inhibition* model and *vicariation* model, the former taking place after small lesions causing mild to moderate motor impairment, the latter observed after larger lesions causing moderate to severe motor impairment (16). The key difference is the competitive vs. cooperative role of the contralesional hemisphere. As a consequence, depending on the severity of motor impairment, therapeutic approaches may prefer to target more uni- or bihemispheric activation, such as when performing unilateral vs. bilateral arm training (17, 18). To date, we are far from having assimilated the knowledge of active ingredients and motor learning principles within each intervention. Coming back to the initial example, for most therapists and clinicians CIMT is somehow synonymous of “the glove”; this is because, in the original CIMT trial, patients had to wear a mitt on the unaffected hand to limit its use during the day, and to remind patients to accomplish functional tasks with the affected hand (19). This vision is reductionist and misleading, considering that the original authors have shown that wearing a mitt is ineffective when tested as isolated ingredient (20); in contrast, other key components of CIMT have shown consistent effectiveness, but unfortunately are still largely neglected.

Therefore, the first objective of the present review was to define each intervention based on key active ingredients, underlying motor learning principles and neural mechanisms likely taking place; the second objective was to draw inferences from research findings, to support our recommendations for clinical practice, familiarize clinicians with the methodology of interpreting evidence, and stimulate further reading and critical appraisal of scientific literature (21). To this end, each chapter is organized with the headers “Intervention,” “Rationale,” “Inferences” and “Recommendations” (see also Table 1). Furthermore, a dedicated chapter on comprehensive assessment of neurological patients is provided, as cornerstone requirement to design a personalized and effective upper limb neurorehabilitation treatment. Finally, we conclude with take-home messages and remarks with direct implications for both clinical practice and research.

**Table 1:** Summary of upper limb neurorehabilitation interventions according to key active ingredients, rationale and recommendations for clinical practice.

**Interventions**	**Key active ingredients**	**Key motor learning principles and neural mechanisms**	**Recommendations**
**Motor training approaches**			
CIMT	Shaping Task practice	Explicit learning Repetitive practice Goal-oriented practice Task-specific practice Interhemispheric inhibition model	Indicated for mild to moderately impaired patients; consider strategies for independent and distributed practice; use of a mitt is not mandatory
Expanded CIMT (adaptated for severely impaired patients) McCabe et al. (22) (intensive rehabilitation for severely impaired patients)	Shaping Task practice Inclusion of orthotics, FES and bilateral training to accomplish tasks	Explicit learning Repetitive practice Goal-oriented practice Task specific practice Vicaration model	Indicated for severely impaired patients; consider devices to assist movement execution; include both unilateral and bilateral arm training
Bobath Concept	Movement quality Afferent input to improve motor control, body schema and motor learning Facilitation techniques (handling, environmental adaptation, verbal cueing)	Explicit learning (implicit feedback/ knowledge of performance) Implicit learning (sensorimotor integration) Task specific practice	Suitable for any level of disability, but currently equally or less effective than other approaches. Consider repetitive practice (high number of repetitions) to increase the effectiveness of the intervention.
**Interventions assisting movement execution**			
Robotic therapy	Repetitive movement performance	Explicit learning Repetitive practice	Particularly useful to assist shoulder and elbow movements (reaching)
Functional electrical stimulation	Repetitive movement performance	Explicit learning Repetitive practice Sensorimotor integration	Particularly useful to assist finger movements (grasping)
splint/orthotics	Repetitive movement performance	Explicit learning Repetitive practice	Particularly useful for wrist and thumb functional positioning
**Interventions providing feedback and multisensory stimulation**			
Virtual reality	Motivation, engagement Visual and auditory stimuli	Explicit learning Explicit feedback Multisensory stimulation	Particularly useful to increase motivation, avoid boredom and promote independent practice. Consider the provision of cues about movement quality (implicit feedback)
Action observation	Immediately after action observation, patients have to perform the same movements with the affected limb	Explicit learning Action observation Mirror neuron network	Indicated for severely impaired patients. Useful as additional therapy time in preparation for motor training
Mirror therapy	Large mirror Unilateral arm performance (unaffected side) No object manipulation	Explicit learning Action observation	Indicated for severely impaired patients. Useful as additional therapy time
Motor imagery	PRACTICE principles	Explicit learning Mental practice	Indicated for severely impaired patients. Useful as additional therapy time in preparation for motor training
**Non-motor interventions priming neuroplasticity**			
Non-invasive brain stimulation	Stimulation parameters and localization of targets	Modulate neural activity Prime motor learning acquisition Interhemispheric inhibition model Vicariation model	Consider different protocols depending on the level of impairment and underlying neural mechanisms. Consider performing motor training during (tDCS) or after (TMS) neuromodulation
Somatosensory electrical stimulation	More than one motor point at the same time, high frequency and intensity near motor threshold	Increased activation of sensorimotor areas Prime motor learning acquisition	Train patients to use the stimulator independently, ideally in the 2 h preceding therapy. Consider providing stimulation concomitant with therapy
Aerobic training	Exercise intensity Performance immediately after motor training session	Prime motor learning consolidation	Consider short high intensity interval training protocols immediately after the motor training session

## Motor training approaches: Constraint-induced movement therapy, bobath concept

### Constraint-induced movement therapy

#### Intervention

CIMT is the most widely investigated motor training approach for upper limb neurorehabilitation, and one of the most effective interventions to date (1, 2, 23). Being originally developed for intensive rehabilitation of patients with mild to moderate motor impairment (19) [bare minimum: 10° wrist extension, 10° thumb abduction, and 10° extension any two other digits (24)], CIMT has been investigated in a number of subsequent trials, although with different doses of therapy (2), and recently adapted to be applicable also for those patients with moderate to severe motor impairment (25). According to the original authors, CIMT is a composite intervention that includes three main elements: restrain of the unaffected hand for the 90% of waking hours, task-oriented training for up to 6 h/day, and a transfer package to foster adherence to the intervention and behavioral changes in the long term (use of logbook, diary, home practice) (5). Within task-oriented training we can further distinguish two components, i.e., task practice and shaping. Task practice is the training of an activity of daily living for about 15–20 min, like preparing a meal, while shaping is the repetitive performance of components of functional tasks, such as reaching and grasping movements (26). A narrative review of Page et al. illustrates practical challenges and opportunities of CIMT, and provides explicit examples to facilitate the implementation of CIMT in a clinical setting (27). Notably, in the adaptation of CIMT for severely impaired patients (“expanded CIMT”), three important ingredients have been the adoption of preparatory techniques for mobilization and tone reduction, the inclusion of bilateral arm training to accomplish tasks, and the use of assistive devices such as orthotics and functional electrical stimulation (25).

#### Rationale

CIMT is a composite motor training approach, where we could identify the systematic application of the following eight motor learning principles: repetitive practice, dosage, task-specific practice, goal-oriented practice, variable practice, increasing difficulty, explicit feedback/knowledge of results, and modulate effector selection. The neural basis of the original CIMT is the interhemispheric competition model, applicable for healthy individuals and patients with relatively small lesions causing mild to moderate impairment (16). According to this model, unimanual activity is the result of the activation of the contralateral hemisphere that sends efferent input to the spinal cord, while at the same time inhibiting the other hemisphere through transcallosal pathways (28). Consequently, the tendency of using only the unaffected arm to accomplish tasks may lead to activation of the unaffected hemisphere and over-inhibition of the affected hemisphere, with negative impact on motor recovery (learned non-use). Therefore, CIMT was designed to restrain the use of the unaffected arm and promote unimanual training of the affected arm. On the other hand, when expanding CIMT to be applicable for severely impaired patients, the vicariation model may be more suitable (16, 29). To summarize, in case of large lesions causing substantial disruption of the corticospinal tract, the brain compensates the lack of resources from primary sensorimotor cortex by recruiting alternative ipsilesional and contralesional pathways to perform unimanual activities. In fact, there are several descending pathways that could subserve motor recovery, arising from parietofrontal regions of both hemispheres and forming direct and indirect (with the interpolation of reticular formation) connections with primary motorneurons of the affected arm (30, 31). Given the cooperative role of the contralesional hemisphere, and the objective difficulty on achieving a functional task with the sole use of the affected arm, the expanded CIMT protocol considered the inclusion of bilateral arm training, less restrictions of the unaffected arm, and systematic adoption of assistive devices (25).

#### Inferences

To understand the relative importance of each component on the final motor outcome, it's useful to compare the effectiveness of CIMT vs. conventional therapy, and the effectiveness of the original CIMT trial vs. modified CIMT versions, generally characterized by reduced time of affected-arm therapy and unaffected-arm restrain, as compared to the original version. Despite the heterogeneity of study designs, patients receiving CIMT consistently showed better outcomes in terms of arm-hand activities and self-reported amount and quality of arm-hand use, than conventional therapy (2). However, there were no significant differences in effect sizes between original and modified CIMT versions (2). Notably, restraint of the unaffected arm and shaping techniques were the two factors applied by any CIMT intervention, but not included in the conventional therapy approaches. Therefore, we can already focus our attention specifically on these two elements.

Restrain of the unaffected arm has been investigated as an isolated intervention, namely “forced use therapy,” but failed to produce any additional benefit on top of conventional therapy, for self-perceived amount and quality of arm-hand use (2). The same authors of the original CIMT compared groups of chronic stroke patients receiving comparable doses of motor therapy (shaping), but different levels of arm-hand restrain, and found no between-group differences in motor outcome (20). According to Taub et al., “there is nothing talismanic about use of a sling, a protective safety mitt, or other constraining device on the less-affected [upper limb]” (32). The point is not wearing a physical restraint, but rather finding a way to counteract the tendency of using exclusively the less-affected arm for all daily activities, which inevitably leads to learned non-use; in this perspective, shaping is in itself already a form of restraint (33). Besides CIMT, a recent meta-analysis (21 studies, 842 subjects) indicated higher improvements for motor impairment, and a trend toward higher functional outcomes, for bilateral arm training as compared to unilateral arm training (34). Such findings challenge the clinical relevance of unilateral training of the affected arm by restraining the unaffected arm, especially if we consider that most activities of daily living require bilateral arm use, and that restraining the use of the unaffected arm is impractical for more severely affected patients. In fact, the expanded CIMT protocol, adapted for those patients with moderate to severe motor impairment, also considered bimanual arm training as part of the intervention, and a reduced time wearing the mitt (25). Moreover, there is accumulating evidence that contralateral arm training, i.e., training of the unaffected arm because of inability to elicit movements on the affected arm, could be beneficial on both the functional and neural domain (35, 36). The mechanism of cross-education (improvement of motor function in the untrained arm following contralateral arm training) may explain this counterintuitive phenomenon. In a recent consensus paper, neurophysiologists and clinicians agreed on that a slight increase in interhemispheric imbalance is acceptable, if function of the affected side is increased following training of the sound limb (37).

Shaping is perhaps the most interesting element of CIMT, surely as a key factor that determined the superiority of any CIMT intervention over conventional therapy, but also because it could be easily implemented in any motor intervention. Shaping can be defined as a structured motor skill training, typically organized in ten 30-s trials for each motor function to be trained, with explicit feedback provided at the end of each trial (5, 26). Movements are organized as discrete repetitions, so that it's possible to train the same pattern of actions consistently and monitor progressions by measuring the number of repetitions performed, the time taken, the distance covered and/or the level of spatial/temporal accuracy while attaining a specific goal. The best performance within each trial is recorded and presented to the patient as reference value, to keep the patient motivated to challenge him-herself on subsequent trials (5, 26). Complex functional tasks (for instance, drinking water from a bottle) are initially broken down into simpler motor components (arm reaching, hand grasping, elbow flexion/extension, etc), until the level that can be practiced effectively and repeatedly by the patient; as the patient progresses, components are gradually practiced in combination, until the whole functional task can be accomplished. The level of difficulty is also adjusted by modulating the required number of repetitions, speed, and accuracy. The selection of the task to be trained is based upon three criteria: (a) being focused on the specific motor impairment shown by the patient (which joints/muscles are involved? which movements are compromised?); (b) being judged by therapists as having the potential to improve the motor outcome of the patient (is there a potential for recovery?); (c) being the task chosen by the patient among a set of similar tasks, in order to meet patient preferences and involve him/her in the decision making process of selecting a task (26). In recent years, high-intensity upper limb rehabilitation trials have consistently reported clinically meaningful motor improvements by applying shaping principles, such as decomposition of functional activities in simpler motor tasks, systematic performance of repetitions and provision of feedback, tasks being specific to motor impairments and toward patient's preferences and goals, and progressive increase of task difficulty in order to meet patient's capabilities and approximate, as much as possible, the functional activity to be trained (22, 38, 39).

An important finding for CIMT and other high-intensity approaches, is the difference in outcomes depending on the stage of recovery. Notably, high intensities of therapy seem more consistently beneficial in the chronic phase (6, 8, 24), than in the acute/sub-acute phase after stroke (27, 28). One possible explanation of such conflicting results is that blocked schedules of intensive training in the early phases post stroke may generate unwanted overload, detrimental for motor recovery and learning (29). It has been suggested that distributing therapy in short sessions throughout the day (spaced practice) might optimize motor learning (28, 30) and even the likelihood of a favorable outcome (31) in the early phases after stroke.

#### Recommendations

1. CIMT is an effective upper limb neurorehabilitation approach that should be embraced by clinicians and adapted to optimally fit with specific patient's needs and resource constraints.

2. Currently, the design of high doses, spaced practice interventions in the acute phase represent a challenge for healthcare providers, and a relatively unexplored, yet promising field of research. Being practically unfeasible for therapists to be present with patients the whole day, soon wearable/portable assistive devices will likely play a key role to achieve high doses, low intensity therapy regimens.

### Bobath concept

#### Intervention

Bobath Concept, or Neurodevelopmental Treatment (NDT), is one of the most popular treatment approaches in neurorehabilitation (4). NDT has been defined as a conceptual framework with the following paramount characteristics: first, the attention upon quality of movement; second, the influence of afferent input on motor control, body schema and motor learning; and finally, the use of facilitation techniques, which includes therapeutic handling, environmental adaptations and verbal cueing (40).

#### Rationale

There are two motor learning principles that are systematically applied within NDT. The first one is task-specific practice: by practicing functional tasks patients focus on learning how to overcome specific limitations in activities of daily living. The second one is the provision of implicit feedback, which could be defined as attention upon movement quality (15). Indeed, a considerable portion of the treatment is dedicated to the improvement of movement quality, by making patients aware of correct movement patterns vs. compensatory and maladaptive movements. A third element is represented by the neural basis underlying “the influence of afferent input” “therapeutic handling,” and “environmental adaptations.” One hypothesis is a multilevel sensorimotor integration (spinal, subcortical, cortical) that is necessary for the initial phases of motor learning acquisition at both conscious (cortical, explicit strategy) and unconscious (spinal, subcortical, implicit recalibration) levels.

#### Inferences

Several systematic reviews, comparing NDT vs. other treatment approaches, have provided substantial evidence that NDT have comparable or even unfavorable effects on several upper limb motor outcomes (1, 3, 4). One reason could be that focusing excessively on activities of daily living and movement quality might come at the expenses of the number of repetitions performed, which negatively impact core motor learning principles like repetitive practice and dosage. Another point of criticism is that NDT historically advised clinicians against engaging patients in resistance training because it may increase muscle tone (41), despite evidence showing that resistance training improves strength and could be beneficial for motor function, without any increase in spasticity (41–43). Finally, there is scant literature documenting the content and theoretical foundations of the Bobath Concept, which is typically taught orally in dedicated courses, thus making NDT prone to detrimental heterogeneity in the interpretation and application of the techniques (4). Recently, promising advancements were the publication of core characteristics of the Bobath Concept (40), and the development of intensive rehabilitation programs based on the Bobath Concept, that led to positive and clinically meaningful improvements of upper limb motor impairment in the subacute phase after stroke (44).

#### Recommendations

We believe that a clearer definition of the intervention protocol, an update of NDT fundamentals based on scientific evidence, and a broader consideration of motor learning principles, with higher emphasis on the volume and intensity of the treatment, might help improve the reproducibility and efficacy of NDT.

## Interventions assisting movement execution: Robotics, functional electrical stimulation, and orthotics

### Interventions

Robotics assist movement execution by providing anti-gravity support and/or by exerting external forces to move joints in a specific direction. Functional electrical stimulation generates movement by triggering internal forces (muscle contraction), with the application of electricity flowing in between pairs of electrodes placed over the muscle belly, at intensities above the motor threshold. For both robotics and functional electrical stimulation, it's preferable that the patient is actively engaged as much as possible in movement performance, with assistive devices set to provide the least amount of assistance to accomplish a certain task. If the patient is unable to perform any movement, they may be used to provide passive mobilization and preserve mobility, to maintain/improve muscle trophism (for functional electrical stimulation), and to normalize muscle tone (45–47). Orthotics provide joint stability in a functional position, which in turn may allow better movement execution and/or increased volume of repetitions for adjacent structures, or at least prevent soft tissue changes secondary to prolonged immobility in vicious postures (contractures, muscle stiffness, joint deformities etc.). Active ingredients are the following: for robotics, we should distinguish between end-effectors vs. exoskeletons, the former leaving more freedom of movement and thus more suitable for mild to moderate impairments, the latter taking control over multi-joint coordination, which is helpful for moderate to severely impaired patients. Other elements to consider is whether the robot allows 2D vs. 3D movements, to which extent it is possible to train proximal and distal components at the same time, or to interact with physical objects. For FES, active ingredients are stimulation parameters, in particular the possibility of eliciting sustained muscle contraction reliably without causing discomfort and pain (48). Furthermore, the possibility of triggering muscle contraction may be preferrable to cyclic stimulation, because of the coupling between electrical stimulation, movement execution and intention to move (49). For orthotics, the possibility to adapt and personalize the device is fundamental to ensure functionality and patient's adherence. Ideal solutions are functional hand splinting made of thermoplastic material, by molding the orthosis directly on patient's hand.

### Rationale

A common goal of assistive devices is the improvement of functionality, either active functionality (better movement quality, higher number of repetitions) and passive functionality (prevention of secondary complications from non-use and immobility). In case of active functionality, the underlying motor learning principle is repetitive task practice enhanced by the adoption of assistive devices. Other motor learning principles may be present, depending on the overall content of the motor training program, the guidance of the therapist and the visual/auditory/tactile/proprioceptive feedback directly associated with the device. Finally, if devices are wearable/portable, this could lead to the application of the principle of spaced practice/distributed practice throughout the day, and increased total dosage of therapy delivered.

### Inferences

Patients with moderate to severe motor impairments require physical assistance to perform active-assisted motor training of the affected limb. Being the distribution of motor impairments rather evenly distributed at the whole upper limb, usually patients require both proximal (trunk, scapula, shoulder, elbow) and distal (wrist, hand, fingers) assistance at the same time (50).

In the simplest form, the patient may use the unaffected arm to support the affected arm, or the therapist may set the scapula in the proper orientation, support the weight of the arm and orient the wrist and the hand in order to perform functional activities, such as reaching and grasping (40, 44). Technological devices may be used as complementary interventions to perform active-assisted arm-hand training, with the therapist having more freedom to provide verbal/physical guidance for correct movement execution, or even without the constant presence of the therapist, to perform additional therapy on top of therapist-patient sessions.

The most extensive upper limb rehabilitation trial provided 300 h of therapy in 5 weeks (5 h/day), and compared the effectiveness of therapist-patient motor learning training, vs. robotics, vs. functional electrical stimulation (FES) (22). Despite being applied on severely impaired patients in the chronic phase after stroke, clinically meaningful improvements in motor impairment were detected for all intervention groups, without any statistical between-group difference. Another large (*n* = 127) although less extensive (36 h in 12 weeks) multicentre rehabilitation trial did not find any clinically meaningful difference between stroke patients receiving equal amounts of intensive robotic and therapist-patient therapy (51). Systematic meta-analysis on the effectiveness of robotic therapy (which included the two aforementioned studies) confirmed that there was no clinically meaningful improvement of motor outcomes, as compared to equal amounts of other interventions (52). Similarly, a systematic review indicated that FES therapy was not superior to other training modalities for the improvements of upper limb motor function (53). Therefore, if the patient performs 100 repetitions of reaching to grasping movements, motor improvements should be rather similar, no matter whether the patient received the assistance from a therapist, a robot, a FES equipment, a combination of these three types of interventions. Notably, most effective interventions considered a composite program, where robotic and/or FES interventions were always performed with various degrees of personnel assistance, and integrated on top of therapist-patient sessions (22, 25, 38, 39, 44). Equal effectiveness between technological and human interventions should be seen as confirmation that motor learning principles, like intensity of training and task specificity, work uniformly across different types of approaches. Therefore, the choice of an intervention over another should depend on factors such as cost-effectiveness, personnel availability, and the opportunity to provide additional training to the patient in group sessions, independently from the therapist, or even remotely.

That said, we should specify that some interventions have more practical applicability for proximal vs. distal upper limb components (22). Assistive devices that showed positive effect on upper limb motor impairment were shoulder-elbow-wrist robotics and wrist-hand-finger FES applications (1). Proximal use of robotics is in line with the fact that most exoskeletons may support as needed the weight of the arm. In general, all antigravity support systems allow patients to perform a wider range of movements, higher number of repetitions, and better movement quality, especially when supervised by a therapist and/or equipped with sensors for feedback on movement performance. A predominantly distal use of FES, on the other hand, is the result of the fact that electrical stimulation is more capable of generating effective movements at the level of wrist and finger into flexion/extension, and to some extent also elbow flexion/extension, than of producing shoulder/trunk movements. The main two variables to consider here are the minimum number of muscles necessary to produce the required movement, and the amount of force that needs to be generated. Elbow/wrist/hand movements into flexion/extension can be elicited by contracting one muscle at a time, eventually providing enough stability to other districts; one example is the application of electrical stimulation of extensor digitorum communis for finger extension, in combination with stabilization of the wrist in neutral position (for instance, with and orthosis). The same cannot be applied so easily to the shoulder, because any movement requires the coordination of a higher number of muscles at the same time, and the control of higher degrees of freedom; for instance, shoulder flexion involves the coordinated activation of the deltoid, rotator cuff muscles, trapezius, serratus anterior and enough core strength.

Finally, if robotics have predominantly a proximal application at the level of the shoulder and the elbow, and FES is effective for finger movements generated by forearm muscles, functional hand splinting at the level of the wrist and the hand may help stabilize the wrist at the desired angle, and recreate a functional shape of the hand as done by intrinsic hand muscles, with a smooth palmar concavity and thumb in opposition, while leaving fingers free to move. All these assistive devices are not mutually exclusive, but rather complementary and have been used simultaneously in composite upper limb neurorehabilitation trials (25, 39) to perform whole arm functional movements, such as combined reaching and grasping tasks.

### Recommendations

Therapists should consider implementing several assistive devices for the rehabilitation of moderate to severe upper limb paresis, within their own treatment plan, to help patients perform functional movements, but also to give “extra hands” to the therapist and leave more space for movement guidance, motivation, and coaching.

## Interventions providing feedback and multisensory stimulation: Virtual reality, action observation, mirror therapy and motor imagery

### Virtual reality

#### Intervention

Virtual reality (VR) has been defined as “the computer-generated simulation of a three-dimensional image or environment that can be interacted with in a seemingly real or physical way by a person using special electronic equipment, such as a helmet with a screen inside or gloves fitted with sensors” (54). Active ingredients that likely play a role are immersive vs. non-immersive VR experience (55); the therapeutic content of the exercise, i.e., being specifically designed for neurorehabilitation purposes (56); the type of feedback being provided (explicit and/or implicit feedback) (57); and the integration of multisensory stimuli (visual, auditory, haptic) (15).

#### Rationale

Motor learning principles that fit well with VR are goal-oriented practice, explicit feedback/knowledge of performance, and multisensory stimulation. Furthermore, because of patient's engagement (by gamification of therapy) and provision of automatic feedback, VR could significantly improve dosage and distribution of training.

#### Inferences

Feedback from a motor act refers to the knowledge of the final result (explicit feedback) or to the knowledge of performance/movement quality (implicit feedback) (15). It's worth noting that the terms explicit and implicit also refer to learning mechanisms, with explicit learning being aware/intentional and implicit learning being unaware/unintentional (58); therefore, the terms differ substantially depending on the argument. Explicit and implicit feedback both subtends explicit learning, with the focus purposely directed toward the end results of a movement (target reached), or how well the movement was performed (coordination, fluency, lack of compensatory movements). A practical example is a patient training forward reaching: explicit feedback consists of the successful grasp of a physical object (a bottle on the table) and positive encouragements from the therapist for task completion; implicit feedback consists on verbal and physical guidance to perform the movement without excessive trunk flexion or shoulder elevation. Feedback is an integral part of learning and has been developed in different forms in any motor training program. There are interventions that have been specifically designed to modulate feedback, such as VR, action observation, mirror therapy and motor imagery.

One modality is to provide enhanced knowledge of result and/or performance during motor skill training, so that the patient can better learn from errors; this strategy is used in VR, for those patients capable of performing a functional task (independently or with the use of assistive devices). Furthermore, an enriched and immersive VR experience, with the provision of different sources of stimuli (visual, auditory haptic) can improve patient's engagement and motivation, which in turn has positive effects on performing higher amounts of therapy, at sustained attention.

A systematic Cochrane review comparing VR vs. conventional therapy found no statistically significant differences on upper limb motor function (59). However, when VR was provided as additional therapy on top of conventional therapy, results were significantly better than conventional therapy in isolation (59). A recent three-arm randomized controlled trial compared high-dose VR, vs. high-dose conventional therapy, vs. conventional therapy; for upper limb motor function, comparable results were found between high-dose VR vs. high-dose conventional therapy, and higher gains in favor of both high-dose groups, as compared to conventional therapy (38). Therefore, one clear advantage of VR is the opportunity to increase the volume of therapy, while at the same time enhancing patient's engagement and sensorimotor integration (60).

#### Recommendations

Movement quality is a cornerstone ingredient of many interventions, by helping patients discriminate between correct and incorrect movements, thus preventing maladaptive learning. Intrinsic feedback gives information about movement quality, and seems to be superior to extrinsic feedback for long-lasting recovery of normal motor patterns (57); notably, to decrease the risk of over-reliance on feedback, a faded schedule should be considered (57). Unfortunately, most VR devices provide a great amount of extrinsic feedback, with intrinsic feedback totally absent or visually presented in the form of mini-avatar in the corner of the screen. In this form, the provision of intrinsic feedback depends upon constant guidance by the therapist; however, if we agree that the greatest advantage of VR is to provide therapist-free additional therapy time, developers of new VR applications should focus on solutions to train the patient independently, with automatic provision of adequate intrinsic feedback, preferably delivered as auditory/haptic feedback, rather than visual (15). As a technical note, wearable VR glasses, integrated with positional sensors of different body segments, might be an ideal starting point in this direction.

### Action observation, mirror therapy and motor imagery

#### Interventions

Action observation typically consists of watching few minutes of video tapes about upper limb functional tasks, performed by healthy subjects; the patient is aware that he/she will practice the same task immediately afterwards, and therapist is constantly present to ensure patient's attention to the video being played, and guide patient in the subsequent task oriented training, for a total of 30–40 min of therapy per day (61, 62).

Mirror therapy consists of placing a mirror vertically at the patient's midline, so that the unaffected side is reflected, thus creating a visual illusion that the image in the mirror corresponds to the affected side. Average training intensity consists of 30 min per day, 5 days a week, for 4 weeks, typically provided as additional therapy to motor training (63). There is a tendency toward better results when using a large mirror, performing unilateral movements with the unaffected arm, without object manipulation (64).

Motor imagery is the mental rehearsal of physical movements (65). Mental practice is structured as a 30-min session, where the patient listens to an audio file in a dedicated, quiet room; the audio file comprises 5 min of progressive relaxation at the beginning and at the end of the session, and 20 min of guided motor imagery (65). Principles of mental practice are part-whole practice with progressive difficulty, repetitive and goal-focused practice, client- and impairment- centered, and with emphasis on task accomplishment (66).

#### Rationale

An alternative approach to VR is to provide patients with a completely fictitious experience of a functional movement, regardless of the actual movement performed/not performed, thus reducing feedback of their own body state. The fictitious experience can be externally (action observation, mirror therapy) or internally (motor imagery) generated; the goal is to activate the mirror neuron system, which has overlapping pattern of activation during action execution, action observation and motor imagery, thus represents a potential way to trigger motor learning and neuroplasticity without even producing a motor action (67); which is particularly appealing for those patients with moderate to severe motor impairment (68). Besides motor learning mechanisms specifically pertaining to these interventions (action observation/embodied practice, motor imagery) another motor learning principle to consider is dosage, because of the possibility to train unassisted/unsupervised, thus increasing the overall volume of therapy performed.

#### Inferences

Several systematic reviews have been already published, overall confirming the effectiveness of these interventions. In particular, a Cochrane review concluded that action observation determines a statistically significant, albeit not clinically meaningful, improvement in upper limb motor function and dependence on activities of daily living, the largest effect being reported for hand function (69). Another Cochrane review found overall moderate-quality evidence that mirror therapy has a positive effect on both upper limb motor function, motor impairment and activities of daily living, though effect size varies depending on the control condition (63). Finally, systematic reviews indicated effectiveness of motor imagery on improving upper limb motor function (1, 23, 70).

#### Recommendations

Action observation, mirror therapy and motor imagery are effective interventions requiring minimal resources, that could be considered as additional therapy to prime motor learning acquisition before the formal patient-therapist session of motor skill training, while virtual reality could be used to increase the amount of active motor training of the affected arm, possibly administered independently from the therapist and in a stimulating, playful way.

## Non-motor interventions priming neuroplasticity: Non-invasive brain stimulation, somatosensory electrical stimulation, aerobic training

Non-invasive brain stimulation (NIBS), somatosensory electrical stimulation and aerobic training are examples of promising interventions targeting biochemical, synaptic and neuroplastic mechanisms underlying motor learning, although they do not consist of any formal motor skill practice, neither active nor imaginative. They are generally provided closely to a motor training session, to enhance motor learning acquisition (immediately before or during practice) and consolidation (immediately after practice).

### Non-invasive brain stimulation

#### Intervention

Non-invasive brain stimulation (NIBS) refers to interventions to modulate neural excitability *via* transcranial stimulation (71). There are two main NIBS modalities, Transcranial Magnetic Stimulation (TMS) and Transcranial Current Stimulation (TCS). Active ingredients are the following: stimulation target, stimulation parameters (intensity, frequency, stimulation protocol), duration, concurrent motor training. International guidelines have been published as reference for researchers and clinicians (72, 73).

#### Rationale

NIBS aims at modulating cortical excitability, either by inhibiting or facilitating neural discharge (74). Regarding upper limb neurorehabilitation, the goal is to prime motor learning acquisition, typically with excitation of the affected sensorimotor cortex and/or inhibition of competitive brain areas before (TMS) or during (TCS) motor training.

#### Inferences

Despite the great potential to enhance the effectiveness of neurorehabilitation interventions, there is a wide variability in response to non-invasive brain stimulation (75). Here we reported four major sources of variability, and provide suggestions on how to account for them:

1) *Severity of motor impairment*. Patients with different levels of motor impairments have quantitative, but also qualitative differences in terms of underlying neural correlates. Quantitatively, the larger the lesion size at the level of the corticospinal tract, the more severe the motor impairment (76, 77). To recap, there are two distinct models of neuroplastic changes following brain lesions: the interhemispheric competition model, where the activation of the unaffected hemisphere over-inhibit the activity of the affected hemisphere, and the vicariation model, where the activation of the unaffected hemisphere is beneficial to partially compensate for the loss of function at the level of the unaffected hemisphere (16, 78). The interhemispheric competition model is valid for patients with relatively small lesions resulting in mild to moderate motor impairments, while the vicariation model is applicable for patients with larger lesions and more severe motor impairments. As a consequence, it is necessary to apply different neuromodulation strategies, depending on the level of motor impairment (16, 79).
2) *The interplay between neuromodulation and motor activity*. Motor activity performed either immediately before, during or after neuromodulation can potentiate its effectiveness, but also have undesired consequences, such as the abolishment or even the reversal of the neuroplastic effects we were initially aiming at (75). Therefore, enough rest time (a few minutes) should be allocated before and after the stimulation protocol.
3) *Stimulation parameters*. Slight modification of stimulation parameters can have relevant and somehow counterintuitive consequences. For instance, increasing the duration of the stimulation protocol reverses the effect of intermittent theta burst stimulation from facilitatory to inhibitory, while continuous theta burst stimulation switches from inhibitory to facilitatory (80, 81). Moreover, increasing stimulation intensity (82) or frequency (83) does not necessarily lead to enhanced effectiveness. Therefore, stimulation parameters should be carefully selected based on evidence of efficacy and within safety recommendations (72).
4) *Aftereffect duration*. Long-lasting effects upon cessation of the stimulation could have important therapeutic consequences, as intensive motor training could be performed in a context of optimal neural state. However, many stimulation protocols have short-lasting effects (< 30–60 min); as mentioned previously, concomitant motor practice or extending the duration of the stimulation are unreliable strategies. One emerging solution is the performance of several stimulation trains within the same day (spaced stimulation) (75, 84). For instance, the application of two trains of continuous theta burst stimulation, with a rest period of 10–15 min in between, has shown long-lasting effects of at least 2 (and up to 10) h (85, 86); four trains of continuous theta burst stimulation (at zero, 15, 60 and 75 min) up to 32 h (87); and finally, eight trains in 2 days produced a clinically meaningful improvement of at least 3 weeks (88). Notably, the outcomes of the studies were the amplitude of motor evoked potentials, saccadic eye latency and visuospatial neglect. Future studies should investigate similar paradigms for motor rehabilitation interventions.

#### Recommendations

Regarding upper limb neurorehabilitation, there is currently contrasting evidence on the effectiveness of non-invasive brain stimulation, with some reviews in favor (89, 90) and others against (91, 92) its added value on top of conventional therapy. Further advances in the field might arise by controlling for factors causing variability in treatment response, and by exploring approaches capable of long-lasting and reliable neuroplastic changes.

### Somatosensory electrical stimulation

#### Intervention

The effect of somatosensory electrical stimulation on upper limb motor function after stroke has been investigated in a number of randomized controlled trials (93–98). Typically, electrical stimulation was administered for 120 min, before the motor intervention, with low frequencies (10 Hz) and intensity above the sensory threshold, but below the motor threshold (99). Only one systematic review (5 studies, 95 subjects) investigated specifically somatosensory electrical stimulation on upper limb motor performance after stroke, demonstrating statistically significant better motor performance in favor of repeated peripheral sensory stimulation (99).

#### Rationale

The objective of somatosensory electrical stimulation is to prime motor learning acquisition, by increasing the activation of sensorimotor areas before or during motor training (100).

#### Inferences

From a theoretical standpoint, it's important to clarify that electrical stimulation should be considered as a pure somatosensory intervention only when the stimulation is above the sensory threshold, but below the motor threshold. In contrast, functional electrical stimulation is a motor intervention, as it requires intensities well above the motor threshold to assist the execution of arm-hand movements. It is unclear whether electrical stimulation at or above the motor threshold, resulting in a visible muscle twitch but no overt movement, should be classified as somatosensory or motor interventions. However, from a clinical perspective, the use of electrical stimulation near the motor threshold level could represent an appealing solution to prime neuroplasticity (101), while not having a practical interference with other motor training interventions performed at the same time, such as motor skill training and motor imagery (102). Indeed, peripheral electrical stimulation induces increased excitability of the corticospinal tract (101), especially when delivered synchronously at more than one motor point (103), at high frequency (≥30 Hz) and at intensities above the motor threshold, sufficient to produce visible muscle twitch or contraction (104). Notably, in healthy individuals the application of electrical stimulation concomitant to motor skill training resulted into higher motor learning indexes, as compared to consecutive sessions of electrical stimulation and motor training (105).

#### Recommendations

There are still several unexplored yet promising applications of electrical stimulation, namely using near-motor threshold intensities, at more than one motor point, and concomitant to other training regimens, that could increase the overall effectiveness of therapy on motor learning and recovery.

### Aerobic training

#### Intervention

The American College of Sports Medicine (ACSM) defines aerobic exercise as any activity recruiting large muscle groups, maintained continuously and rhythmic in nature (106). Active ingredients could be defined according to the following variables: training modality (cyclic movements vs. discrete repetitions), training intensity, duration, inter-session intervals and overall intensity and volume of the program, in terms of frequency of sessions per week and total number of weeks.

#### Rationale

The main goal of aerobic training, when performed immediately after motor skill training, is to enhance motor learning consolidation (107–109).

#### Inferences

Motor memory encoding and consolidation are two consecutive phases of the motor learning process. In healthy individuals, aerobic exercise enhances motor memory consolidation, but not acquisition, with strongest effects shown for high intensity exercises (107). Aerobic training performed immediately after motor skill training improves sleep-dependent consolidation (107), and leads to higher effects on long-term retention than when performed immediately before motor skill training, or when aerobic training is not performed (108). In line with these findings, patients in the chronic phase after stroke showed better retention when 15 min of high intensity interval training (HIIT) was performed immediately after motor skill training, as compared when motor skill training was followed by a period of rest (109). HIIT consists of repeated, short bouts of exercise at elevated effort, interspersed by periods of low-intensity exercise or rest. In the rehabilitation field, the main attractiveness for HIIT is that results, even for deconditioned patients, in cumulatively more time of exercise at high intensity and lower perceived effort, compared to moderate-intensity continuous aerobic training (110). A recent review indicated the potentialities of HIIT, as safe and effective intervention to promote cardiovascular, functional and neuroplastic outcomes post stroke, but at the same time highlighted that larger randomized trials are needed to confirm findings from pilot studies (111). It's important to highlight that there are no specific safety issues for HIIT, as compared to moderate intensity continuous cardiovascular exercise (111).

#### Recommendations

There is international consensus that, once the patient is medically stable and cardiopulmonary exercise testing has been performed to rule out risks of adverse events, an aerobic training program should be implemented in the general rehabilitation program (112, 113); the beneficial effects on cardiovascular status, functional recovery and motor learning are directly associated with the intensity of the exercise so that, the higher the intensity, the higher and enduring the therapeutic gains (111, 114).

## From comprehensive assessment to personalized interventions

Personalized, patient-centered goal setting is perceived important by stroke survivors and caregivers, but this practice is often lacking (115). Tailoring neurorehabilitation to individual needs and preferences, monitoring progressions, and maintaining a positive and proactive attitude toward realistic goals, is important to improve motivation, adherence, effectiveness and patient's satisfaction of the treatment received (116). The foundation of personalized interventions is a comprehensive assessment of both objective impairment and patient's perspective, and the definition of shared goals and expectations between the patient and the therapist.

**Figure 1:**
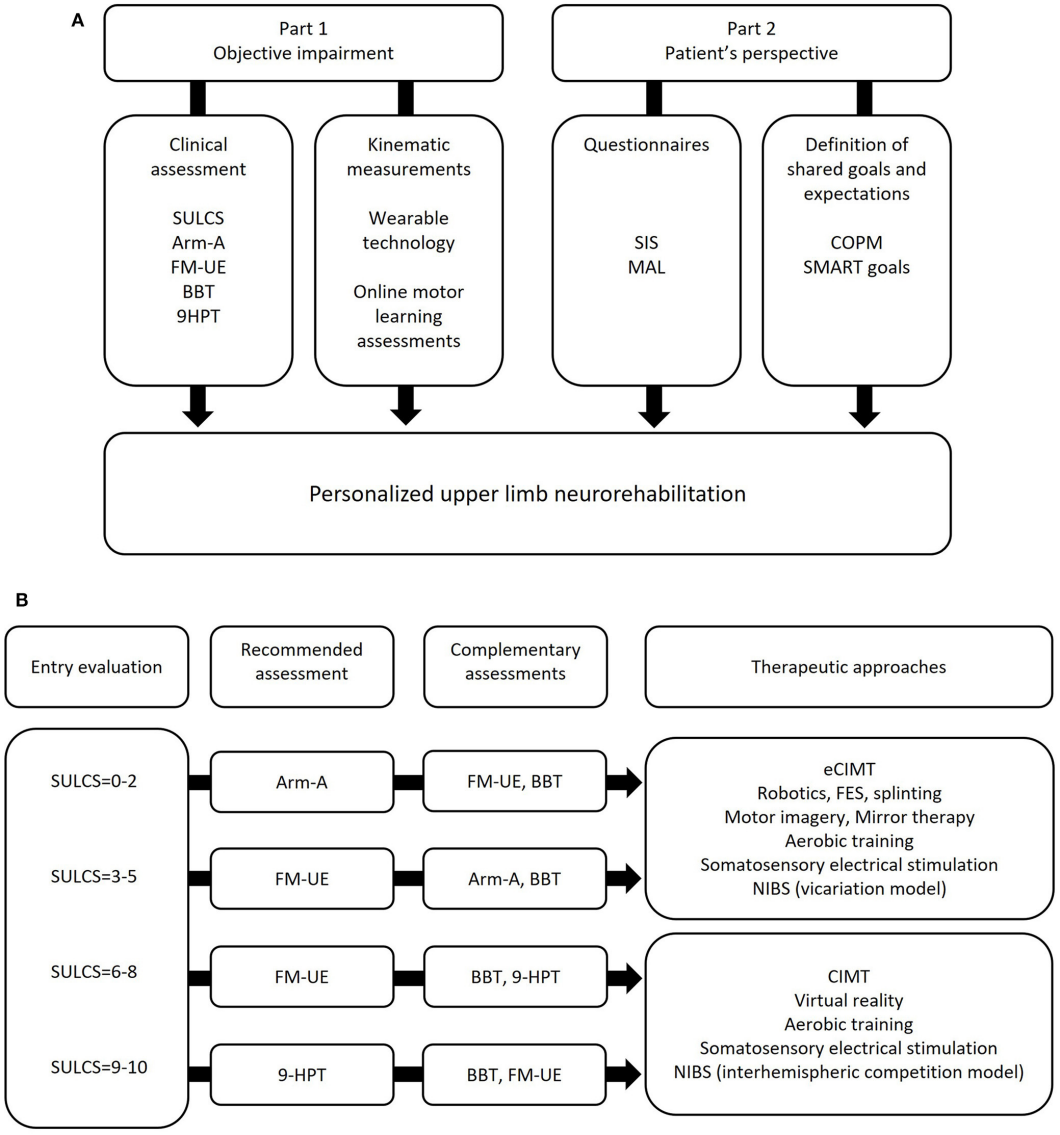
**(A)** Schematic representation of comprehensive upper limb neurorehabilitation assessment; **(B)** example of assessment algorithm for objective upper limb neurological deficits, developed and applied at Guttmann Institute (Barcelona, Spain). 9-HPT: 9-Hole Peg Test; Arm-A: Arm Activity Measure, section A; BBT: Box and Block Test; CIMT: Constraint Induced Movement Therapy; eCIMT: expanded CIMT; FM-UE: Fugl-Meyer Upper Extremity assessment; NIBS: Non-Invasive Brain Stimulation; SULCS: Stroke Upper Limb Capacity Scale.

Figure 1A illustrates a schematic of the proposed workflow. The first step is to determine objective deficits, for instance by using validated clinical assessments covering the ICF domains of body function and structure (impairments) and activity (limitations) (117). The second step is to consider patient's perspective to quantify real-life perceived functionality (validated questionnaires) and to determine goals for treatment. However, sometimes it's difficult for patients to identify their own functional goals (27). To facilitate the decision-making process, it's recommended to use semi-structured interviews such as for the Canadian Occupational Performance Measure (118), and SMART principles for patient-centered goal setting (119). Finally, there is growing interest in the development of wearable sensors and remote assessments to automatically and reliably monitor real-life functional use of the affected arm (120–122). One of the greatest advantages of tele-assessment is that it may be diriment to interpret the potential gap between what is objectively measured in the clinic, and how patient perceives arm functionality (123, 124).

Figure 1B provides a practical example of clinical assessments to determine objective upper limb deficits and select appropriate treatment strategies. The criteria for selecting each assessment measure where the following: (1) well-established psychometric properties (validity, reliability, sensitivity to change); (2) clinical utility, meaning the ability to already direct therapists toward concrete therapeutic goals; (3) easiness of use, in terms of training and equipment required, time to perform the assessment, and potential contraindications. For instance, the Stroke Upper Limb Capacity Scale (SULCS) is a clinical assessment of upper limb activity, with excellent inter-rater reliability and high responsiveness to change (125–127); there is minimum training and equipment required; tasks are organized hierarchically, so that therapists may decide to start from the beginning or from the end of the assessment, and to progress until upper limb functionality have been determined (no need to perform all the tasks), which is time-saving and avoids patient's frustration or boredom for too difficult or too easy tasks; finally, tasks cover common functional activities, like drinking from a glass or fastening buttons, which could already indicate goals for therapy. For these reasons, SULCS was considered as quick and useful entry evaluation; SULCS score may be used as indication for further assessments (either recommended and complementary) and therapeutic options. The present assessment algorithm has been developed at the Guttmann Institute (Barcelona, Spain) by physiotherapists and occupational therapists, and is currently being used in the clinic as structured pipeline to evaluate upper limb neurological deficits.

## Conclusions and future perspectives

Upper limb neurorehabilitation is a difficult field of intervention, that keeps challenging specialized healthcare providers to find tangible solutions to improve patients' motor outcomes and quality of life. The significant advancements of the last years are the result of a collective, international effort from thousands of passionate researchers and clinicians. It's now essential to put research findings into practice, to provide patients with the best opportunities of regaining functionality and independency.

For the sake of synthesis, we try to provide a list of specific recommendations for clinical practice, technological developments, and future research on upper limb neurorehabilitation:

- Patient-therapist session: consider two main phases: shaping and task practice. Shaping has the main goal of performing a great number of repetitions in a short time frame, typically singular components of a functional task, preferably trained in an engaging, variable, and challenging way; enough time should be dedicated to this section, at least half of the total therapy time. Task practice should be performed as the final part of the session, to capitalize what was done in the previous phase for the relearning of a whole arm functional activity. A third phase that was not mentioned previously in this article but nonetheless should be considered, is preparation. Preparation means daily briefing, body awareness and physical techniques to ensure proper stability of the proximal segments and mobility of the distal components, before shaping and task practice; preparation might require more time at the beginning of the rehabilitation program but should be gradually reduced as soon as the patient progresses, and in any case contained within one third (or less) of the total therapy time, to prioritize motor practice.
- Before/during motor task practice: consider motor imagery and somatosensory electrical stimulation to prime motor learning acquisition. For motor imagery, consider the development of audio tracks that patients can listen to while resting in a quiet room. If you are using a FES device during the patient-therapist session, consider the possibility of modulating the intensity of the stimulation above the motor threshold for movement production, but remaining between the sensory and motor threshold during rest periods.
- After motor task practice: consider aerobic training to enhance motor learning consolidation, and for cardiovascular benefits.
- Robotics and FES: developers should consider the creation of devices with both proximal and distal effectors, or hybrid devices (robotic/proximal and FES/distal) for the performance of reaching and grasping motor training.
- Virtual reality: developers should prioritize the automatization for the provision of intrinsic feedback, preferably as haptic/auditory feedback.
- For any intervention: clinicians, technological developers and researchers should find therapeutic solutions that allow patients to train unassisted and unsupervised. The addition of independent therapy time *on top* of the formal patient-therapist session may represent the leading strategy to provide high volume of training, distributed throughout the day, and economically sustainable in the long term.
- Personalized interventions: perform a comprehensive evaluation of both objective neurological deficits and patient's perspective to develop tailored therapeutic goals and personalized interventions.

## Author contributions

LB and JT conceived the original idea and drafted the first version of the manuscript. All authors provided substancial contributions and approved the submitted version.

## Funding

The authors affiliated to Institut Guttmann disclosed receipt of the following financial supports for the research, authorship, and publication of this article: Programa Joan Ribas Araquistain de Investigación, Innovación Terapéutica en Prehabilitación, Rehabilitación, Abordaje integral de las secuelas de Tumores cerebrales from Fundaciò Joan Ribas Araquistain (Reference Project 2020.330), Assaig controlat aleatori de l'efecte potenciador de l'estimulació transcranial de soroll aleatori (tRNS) en la rehabilitació cognitiva dels pacients amb lesió cerebral traumàtica from Fundaciò La Maratò De TV3 Convocatòria d'ajuts projectes de recerca en Ictus i Lesions Medullars i Cerebrals traumàtiques 2017 (reference project 201735.10), BBHI, Barcelona Brain Health Initiative from Fundaciò Bancària La Caixa.

## Conflict of interest

The authors declare that the research was conducted in the absence of any commercial or financial relationships that could be construed as a potential conflict of interest.

## Publisher's note

All claims expressed in this article are solely those of the authors and do not necessarily represent those of their affiliated organizations, or those of the publisher, the editors and the reviewers. Any product that may be evaluated in this article, or claim that may be made by its manufacturer, is not guaranteed or endorsed by the publisher.
